# "The Hospital" Nursing Mirror

**Published:** 1900-06-30

**Authors:** 


### "The Hospital" Nursing Mirror.

Being the Nursing Section of "The Hospital."

[Contributions for this Section of "The Hospital" should be addressed to the Editor, The Hospital, 28 & 29, Southampton Street, Strand, London, W.C., and should have the word "Nursing" plainly written in left-hand top corner of the envelope.]

### Notes on News from the Nursing World.

### THE QUEEN AND THE MATRON OF WINDSOR INFIRMARY.

It is characteristic of Her Majesty that directly she returned to Windsor she paid a visit to the Royal Infirmary, and sent an attendant to ask Miss Macdonald, the matron, to come out and see her for the purpose of affording her information concerning the sufferers from the collision at Slough. When this had been given, the Queen made kindly inquiries as to how the staff of the infirmary, which she knows is none too large, managed with so many cases. The matron explained that some of the patients already in the hospital had been removed to another institution to make room for the new comers. Since her visit the Sovereign has continued to manifest the utmost concern for the sufferers, and on Saturday sent them a quantity of fine grapes and the freshest strawberries from the Royal gardens at Frogmore. The fruit was distributed by the matron and nurses among the ten patients, each of whom received a basket of strawberries and a plate of grapes.

### ANOTHER DOLE OF NURSES FOR THE FRONT.

The Secretary of State for War informs us that on Saturday the following 20 sisters of the Army Nursing Service Reserve will embark for South Africa: Nurses M. A. Blair, S. Brown, M. E. Buse, A. E. Byrne, S. A. Claridge, A. D. Cameron, E. P. Carruthers, C. Condell, G. Deacon, F. Donald, C. D. Elmslie, M. A. C. Grant, M. M. McIntosh, E. McIntosh, A. M. McIntosh, M. A. C. Millington, K. M. Picot, J. E. Phillips, G. L. Shelley, and A. A. S. Webster. Miss Ann Eliza Byrne was trained at Cardiff Infirmary, where she was subsequently staff nurse. She has also been attached to the Private Nursing Institute at Swansea, and since October, 1892, has been engaged in private nursing at Cardiff. Miss Grace Lillian Shelley was trained at the Guest Hospital, Dudley, and has since been charge nurse at the Hospital of St. Cross, Rugby, from January, 1896, to January, 1898; and staff nurse at the General Hospital, Birmingham, from January, 1898, to the present time.

### PENSIONS OF ARMY NURSES.

On Friday evening Mr. Griffith Boscawen asked the Under Secretary of State for War whether his attention had been called to the scale of pensions given to members of the Army Nursing Service, under which the highest pension that an army nursing sister can receive is £37 a year after 21 years' service, that she cannot receive more even if she continues to work till she is 60 years of age, and that the average pension is less than this. The member for the Tunbridge Division also asked whether, in view of the fact that all the members are required by the regulations to be ladies of good standing in society, and that they have done the most excellent work, especially in South Africa during the war, pensions on a more liberal scale could be provided. It cannot be said that the answer he received to his questions was satisfactory. The Under Secretary declined to make any concession whatever. But he pointed out that a sister can get a pension of £50 per annum for exceptional devotion to duty; and from the tone in which this was said, Mr. Griffith Boscawen himself gathers that such additional pension might be granted to any nursing sister who has done good work in South Africa. At all events, if any such application is made to the War Office, he will be glad to support it.

### COMFORTS FOR NURSES AT THE FRONT.

The nurses at the front know that they have warm friends among the public at home, but we do not doubt that every practical evidence of sympathy will be warmly appreciated. Mrs. Leather-Culley, who is starting to South Africa on Saturday, "feels that the nurses must often wish there were shops at the front instead of only the bare veldt," and she is taking out with her a number of articles likely to be wanted badly. Her self-imposed mission is approved by the Royal Army Medical Corps. The articles entrusted to her she will place, on her arrival, at the disposal of Sir John Furley, Chief Commissioner of the British Red Cross Society, and we learn that she has received "general and generous support." It is not suggested that the nurses cannot afford to purchase the articles in question, but the idea is to show a little gratitude for their professional services to our soldiers by providing them with things which they may not be able to obtain where they are.

### ARTICLES WANTED FOR THE WOUNDED.

Some time ago we alluded to the zeal and energy of the nurses and patients of the Samaritan Free Hospital in making and sending 80 cushions to the De Aar Hospital. The following letter to the matron not only shows what a boon the gift has proved, but it offers some guidance to others anxious to assuage the sufferings of the sick:—

"Office Soldiers' Comforts, c/o Major Cresswell Clark, Traffic Manager, De Aar Station, May 21st, 1900.

"Dear Madam,—I beg to acknowledge with thanks the receipt of your presents for the soldiers, which, owing to the great need of trucks, have only just arrived. The 80 pillows are most acceptable, and will be greatly appreciated. Please tell your friends that underwear, towels, socks, cholera belts, woollen caps, soap, tobacco, pipes, small note-books, indelible pencils, and small bags for carrying extra kit, are the most urgent wants of 'Tommy.'—Yours gratefully,

"Jennie Clark.

"To Miss Butler, matron, the Samaritan Hospital, Marylebone Road, London."

### SISTER ROSE STANLEY.

The nurse who was injured in the railway collision at Slough was Mrs. Stanley. She went out to Johannesburg some time ago and there married Mr. E. L. Stanley. After the war broke out they both found themselves refugees at Durban, where the husband volunteered for Bethune's force, and Sister Rose Stanley volunteered for hospital work at the front. She was placed in charge of a temporary hospital at the vicarage at Greytown, where her first patient was her own husband, who, as a victim to enteric fever, she nursed for five weeks. Afterwards she was placed as nurse in charge of the wounded in troopships from Durban to Cape Town. She came home in a troopship with wounded soldiers.

### NURSING IN FEVER HOSPITALS.

Dr. Goodall, medical superintendent of the Eastern Hospital, states that the chief difficulty with respect to the staff has been, and still is, the question of obtaining and keeping a complete and suitable staff of nurses. There is, even in this respect, an improvement, for Dr. Goodall observes that "formerly it was difficult to get good nurses at all." The opportunity which is taken in the slack season to let the nurses have their holidays has, however, effected an improvement, and he now finds that, so far as the Metropolitan Asylums Board hospitals are concerned, this difficulty is confined to the first-class assistant nurses. "Many of the charge nurses," he goes on to say, "have a good deal to learn after entering upon their duties." It is true that no charge nurse is engaged before she has produced her certificate of three years' training in a recognised training school for nurses; but Dr. Goodall's experience is that "many nurses go through their whole period of training without ever having nursed a tracheotomy case, syringed out a throat, or passed a nasal tube." Also that "on matters relating to infection they are often much below the standard of knowledge required of a charge nurse in a fever hospital." Then, "when they have received instruction on these points, have gained sufficient experience to enable them to undertake the nursing of fever cases in private, and have served long enough to be entitled to a testimonial, off they go." The result is that the nursing staff is constantly changing. But he admits that a fever hospital cannot expect to attract to its service in any large numbers women of the stamp nowadays to be found in the best general hospitals, because the latter offer a training in most, if not all, branches of nursing; because of the social ostracism involved in life in a fever hospital; and because of the fear of contracting disease. As to the last, Dr. Goodall remarks: "This is no idle fear. During 1898, of 4,192 persons employed in the fever hospitals of the Asylums Board 1,449 were 'warded off' on account of illness, and 8 died."

### VICTORIAN ORDER OF NURSES FOR CANADA.

Progress marks the career of the Victorian Order of Nurses for Canada. In the annual report of the Board of Governors it is stated that, as the result of a tour by Miss Macleod, a nurse has been sent to North Bay, Port Arthur, Fort William, Thessalon, Fort Frances, and Bracebridge; and the hope is expressed that, after a year's experience of the presence and services of the nurses, these stations may become, to a large extent, self-supporting. Three new local associations have been constituted during the year—at Kingston, Ontario; Hamilton, Ontario; and St. John, New Brunswick. The nurses of the Victorian Order have been withdrawn from the Klondike, where district nursing cannot, it seems, be carried on with advantage, and hospital work is alone available. An item of personal interest is that Miss Bertha Hall, of Montreal, who has been in the service of the Queen's Jubilee Institute for Nurses in England, and is therefore a Queen's nurse, has been appointed head nurse at the Ottawa Home. There are now twenty-five fully-qualified nurses in the service of the Victorian Order, and seven undergoing training in district nursing as probationers for the Order.

### THE REVOLUTION AT SINGAPORE GENERAL HOSPITAL.

A great change—in fact, nothing less than a revolution—has been made in the nursing staff at the Singapore General Hospital. The 12 French sisters, who for years have constituted the staff, have left in a body, and their places have been taken by half-a-dozen trained English nurses. There has been no neglect of duty on the part of the French ladies, who always did their best for their patients, and whose kindheartedness and devotion have never been called in question. But the new policy was decided upon solely with the view of affording the patients the advantage of a system of proper trained nursing. The French sisters return to the convent at Singapore, and when the six English nurses have got things into working order, local probationers will be taken at the hospital, and trained with the object of enabling them to act as nurses. The change is one of obvious importance, and, it may be hoped, will be the precursor of a similar alteration in many other Colonial hospitals.

### THE PRINCESS KOMATSU OF JAPAN AND THE "LONDON" HOSPITAL BONNETS.

The Princess Komatsu, who is the head of the Japanese Ladies' Nursing Guild, was so pleased with the "London" Hospital bonnet worn by one of the nurses who has gone out to Japan under the auspices of the Colonial Nursing Association, that she has sent it to Yokohama to be copied. In future all members of the guild are to wear bonnets when they are on duty.

### A PLEASANT ENTERTAINMENT TO NURSES.

Last week, on the invitation of Mrs. G. Danford Thomas, the matron, night sisters, several sisters, and a few of the senior nurses of St. Mary's Hospital had an early morning drive to Foley House, Hampstead Heath. The coach left the hospital at ten minutes past six, and the party proceeded as far as the Welsh Harp, Hendon, where they were met by Dr. Danford Thomas, and regaled with tea. At Golder's Hill they were courteously received by the matron of the Convalescent Home for Wounded Soldiers. The patients were at breakfast, and seemed very cheerful and contented. Our correspondent describes the reading-room as the picture of comfort, with plenty of easy chairs and nice soft cushions. There were only two men in bed. At Foley House, where Mrs. Danford Thomas and her family gave the party a hearty welcome, a sumptuous breakfast was served, and ample justice was done to it. After breakfast the guests took a walk in the garden, and subsequently returned to their work strengthened and refreshed by a most delightful outing.

### A DRAWBACK TO NURSING IN INDIA.

"A nurse in India" writes: "One experience I came across in our wards I hope is rare. We have a man in for enteric—one of many; their tongues become very furred and bad, but never before did I know of a patient breeding maggots in his mouth. The medical officer has come across similar cases. The cause is the dreadful flies who buzz round, and in a man's mouth, when open, lay their eggs. The Government have offered a prize to anyone who will rid the wards of these flies. Of course, they fasten on us who are always round the beds of the patients. We have to syringe out the mouths of the latter. My patient actually had holes in his top and lower jaw inside made by these dreadful insects."

### CANON FLEMING ON FLORENCE NIGHTINGALE.

There were, of course, many services preached last Sunday on behalf of the hospitals, though some of the clergy contented themselves by merely announcing that the offertories would be on behalf of the Hospital Sunday Fund. At St. Michael's, Chester Square, which always occupies a first place in the list of contributories to the Fund, Canon Fleming, taking for his subject the parable of the good Samaritan, said, "There have been women more brilliant than Florence Nightingale, yet all parts of the world sang her praises when she reached her eightieth birthday, and that army of nurses who are working out in South Africa are her disciples and her children."

### WINIFRED HOUSE CONVALESCENT NURSING HOME.

The annual meeting of the supporters of the Winifred House Invalid Children's Convalescent Nursing Home, Tollington Park, was held on Monday evening at University Hall, Gordon Square, Mrs. Fawcett in the chair. After the hon. treasurer, Mr. W. M. Blyth, had read the financial statement, which showed a decrease in the annual subscriptions, and stated that £250 in Consols had been sold out to meet the expenses of re-drainage, Mrs. Fawcett moved the adoption of the report. She spoke of the excellence of the work which is being done at Winifred House. It was built in 1881 for the purpose of carrying on the work then known by the name of Mrs. Hampson's Home, and to it are admitted invalid children who are recovering from disease after treatment, either in hospital or at home, and who only need careful nursing, under medical supervision, wholesome food, and fresh air in order to complete their restoration to health. Suffering, she said, had been spared, and lives prolonged by means of the work, and the home had been an immense help to parents of the children in times of trial and difficulty. It is a feature of the home that the length of stay by most of the invalids is protracted, and last year, owing to this and to the fact that the building was closed for nearly eight weeks for drainage reconstruction, only 33 children were admitted.

### MISS DOWLING'S LIBEL ACTION.

Although the jury last week decided the case of Dowling v. Dods in favour of the plaintiff, execution has been stayed by Mr. Justice Darling on the ground that the verdict was against the weight of evidence. Miss Dowling, who is described as a certificated nurse, brought an action against Mr. Louis F. Dods, a surgeon in Marylebone Road, for alleging that she was of unsound mind, and the jury in the Court of Queen's Bench awarded her £100 damages. But this amount she will not, of course, receive unless or until the verdict is confirmed by the Court of Appeal.

### PERSONALITIES AT THE MEETING OF A LINCOLNSHIRE INSTITUTION.

A very discreditable state of affairs seems to prevail at Scunthorpe, in Lincolnshire. At an extraordinary general meeting of the members of the local nursing institution it was proposed by Mr. J. A. Cutts that the institution, as constituted at present, should be disbanded. Whereupon, according to the *Boston Independent*, the following scene occurred:—

"Mr. A. Thurston moved an amendment to the effect that the rules be altered. Mr. Poole said there was an under-current of bad feeling which was not for the good of the institution, and it would have to be eliminated. Dr. Behrendt said there had been a lot of malicious tittle-tattle gossip taken to the lady superintendent, and that has been the ruin of the institution. Nurse Dosser charged the Chairman (the Rev. S. Cutts) with misrepresentation of a private interview with her. The Chairman warmly denied the charge. The Nurse, continuing, said she objected to the social position of the new lady secretary—her social position was not good enough for her to be put over her (the nurse). The Chairman termed this a downright insult—he never heard a worse one. The nurse had impeached his Christianity and veracity. The Rev. J. Keightly said they ought to dig a grave and bury everything unpleasant, and not vote on the matter under the influence of passion. Mr. J. A. Cutts thought that they could not do anything until they got rid of the present nurse. Mr. Thurston said Mr. Cutts' remarks were abominable, and a direct insult to the nurse. Mr. J. A. Cutts' motion was lost, whereupon the Rev. S. Cutts said he could not retain his position as chairman of the institution, and withdrew from the chair. It was decided to call a special meeting to revise the rules."

But a more drastic alteration than a mere revision of rules seems requisite to meet this case. The manners of some of the members might be revised with advantage.

### SHORT ITEMS.

At the general meeting of the Japanese Red Cross Society last month, in the Palace of Prince Komatsu, it was resolved to send the special medal of the society to Miss Florence Nightingale.—The second annual meeting of the London School Nurses' Society will take place on Friday, July 6th, at five p.m., at the School Board Offices, Victoria Embankment. The Countess of Aberdeen will take the chair, and the Bishop of London will be amongst the speakers. All nurses will be welcome.—The Mansfield Board of Guardians has given £21 to the District Nursing Association. Last year the nurses visited 355 cases; paying 8,564 visits.—The annual meeting of the Central Bureau for the Employment of Women will take place at Queen's Hall on Thursday, July 5th, at half-past three p.m. The chair will be taken by Adeline Duchess of Bedford.—A correspondent asks us to say that at the reception of Glasgow nurses by Princess Christian Mrs. Strong and Mrs. Harbin wore silver medals, and Miss McFarlane and Miss Husband bronze medals of the R.B.N.A. The Town's Hospital, Glasgow, to which no reference was made last week, was represented by the assistant lady superintendent, Miss Finn, and one of the charge nurses.—A public conference on hospital and nursing affairs is to be held, under the authority of the Matron's Council, at the Medical Societies' Rooms, 11, Chandos Street, Cavendish Square, Thursday and Friday next week, at three p.m. Miss Isla Stewart will preside, and the following questions will come under discussion: "A Poor Law Nursing Service," "The Necessity for a Nursing Department in all Governmental Offices dealing with the Nursing of the Sick," "The Celebration by Trained Nurses of the New Century," "The Nursing of the Sick at Sea," and "The State Registration of Trained Nurses."—Miss Redmayne, whose appointment as staff nurse at Victoria Hospital, Woking, was announced last week, states that she was on the private nursing staff of the Newark Hospital in 1896, and has not worked at the Branch Seamen's Hospital.—A very pretty wedding took place in Shrewsbury on Saturday, when Miss M. A. Kincey, of the Nursing Institution, was married to Mr. C. E. Roden, of Broseley, at St. Mary's Church, by the Vicar.

### Lectures on Medicine to Nurses.

By H. A. LATIMER, M.D. (Dunelm), M.R.C.S. Eng., L.S.A.Lond.; Consulting Surgeon, Swansea Hospital; Past President of the South Wales and Monmouthshire Branch of Brit. Med. Assoc.; Past President of the Swansea Medical Society; Hon. Life Member of the St. John Ambulance Association, &c.

### THE MODERN NURSE CONTRASTED WITH THE NURSE OF THE PAST.

As an essential and highly valued part of modern progress in the treatment of disease stands the work of the trained nurse, whose co-operation with the medical man in the care of the sick has proved of inestimable advantage to all concerned. What a change has taken place in this regard during the past two or three decades! I do not know how long many of my readers have been engaged in the vocation of nursing, but I presume that the majority of them have come into the ranks of their profession in recent years; if such should prove to be the case they can have little conception of the state of their calling in the pre-scientific days of only a few years ago. It is true that in the great hospitals of the country the nursing of sick people has been carried out with a certain degree of efficiency for many years past, but even there the work was performed with only the training in mechanical matters which constitute the actual work of attendance on the patients under a nurse's charge. It is only of comparatively recent date that the trained nurse has undergone a systematic instruction which embraces the study of a sufficiency of anatomy, physiology, medicine, and surgery to enable her to understand the aims and objects of the treatment of disease by medical men, as well as systematic training in the wards of hospitals, in the practical duties of her calling at the bedside. Some of you, I have no doubt, have groaned at times over the large amount of work which you have had to do when you have been under training; it has been a weariness to the flesh to attend lectures and to study theoretical subjects when you have been fatigued with ward work. The cry has arisen, *cui bono?* What, you may have said, has all this theory which I have to master got to do with the practical work of a nurse; can it be necessary for her to have all this training in the rudiments of some sciences to enable her to attend the sick? I know that such questions as these arise in people's minds, and I know what an answer can be given to them. The answer presents itself when the modern highly-trained nurse comes on the scene, and when her demeanour and work are contrasted with the demeanour and work of the nurse of pre-scientific days. There were then, as there always has been and I hope ever will be; women, who by natural aptitude, made splendid nurses—affectionate by disposition, sympathetic with suffering, gentle in touch, and quiet in their movements. Such attendants in the sick-room were a blessing to those who needed their ministrations. But they had, as it were, to grope their way to knowledge, and such helpful people were to be met with but rarely, the work being for the most part performed by persons who were unreliable, ignorant, and prejudiced to a singular extent. I do not care what the vocation in life may be: whatever it is, the trained person who exercises it must (if the training be good and the opportunity afforded by it of mastering the art which is being studied is made proper use of) rise superior to the untrained one. The fact applies to nursing as it does to other callings. Before this became a specialised calling, with systematic training at the beginning of the career, it was too often the case that people only became nurses when, from some cause or other, they had to seek about about for some means of making a livelihood. It was not then the general rule to engage a nurse during illness. Unlike what obtains now, when one of the first thoughts of the medical attendant and the friends when any illness declares itself to be of a serious nature, is that a trained nurse must be secured, it was too often the last idea in the rôle of treatment to be considered. And thus, when the nurse of those days was brought into the sick room, she often did little more than keep the fire going, administer medicines, make the bed, and gossip. In dread of such attendance the nursing of the invalid was generally undertaken by some one or more of the family, with the by no means rare result that one or two more patients appeared on the scene beside the original one, for the doctor to attend, when the amateurs had broken down in health from overwork and over-anxiety.

And, oh, what a tangle and a bother the untrained and undisciplined nurse of the past has often proved herself to be. I have been long enough in the medical profession to have been brought into contact with her, and to have suffered in patience and in temper on her account. For in proportion with her ignorance she was wedded to theories and practices of her own which were generally to the detriment of her patient's health. If you wanted to see her in the full perfection of her misdeeds you would meet her in midwifery practice. Here she had as a rule for her only qualifications —I speak now of the midwifery nurse who attended the less well-to-do people in the community—the oft-proclaimed fact that she had been the mother of a family; this, in her own eyes, constituted her a perfect attendant upon a lying-in woman. She was possessed of a number of ancient maxims which were her rule in life, and if she could circumvent the doctor, when his back was turned, in the carrying out of any of these theories she would do so. She would squeeze the newly-born baby's nipples to "break the strings" if she could, thereby provoking many an abscess in the breast. Unless sharply looked after she would not change any body-linen or bed-linen of the patient's until three days had passed by, in that way provoking many an attack of puerperal fever; and when the fever was established she would always attribute it to some slight and accidental exposure of the patient. An open window in the sick room was an abomination in her eyes, and fresh air, in her opinion, was provocative of any amount of chills and illnesses. Being ignorant, shiverings and rigors were always put down by her to colds having been caught; that they could possibly be the outcome of a poisoned condition of the blood was not to be thought of for one instant. I am thankful to say that this class of person is rapidly disappearing, her place being taken by the well-dressed, neat, cleanly, and skilful modern-trained nurse. I used to be heartsick in the old days, when attending the poor, when I thought of the mischief wrought amongst them by the neighbours who posed as nurses during illness; for not only did they intrude with harmful medicines when my back was turned, but they were pretty sure to wilfully disobey directions which had been given them, and to vow, when taxed with it, either that the patient would not allow the treatment to be carried out, or that all that I had ordered had been obeyed to the letter. Contrast such a state of things with what is taking place now, and you will see what improvements have taken place. The modern nurse, having received instruction in hospitals both in the technical and in the theoretical parts of her duties, understands the why and the wherefore of the doctor's orders, and, having learnt that she should loyally carry out his instructions, may be generally relied upon to co-operate with him for the welfare of her charge. She does not want to "score off her own bat" to his disadvantage; it is enough for her that by doing as she has been directed, and by sympathetically performing the duties of her office, she will get the full credit which she deserves for her work. Of one thing I am sure, as the result of experience: if she takes the view of her office which I have been describing above, she never fails to be valued and to be liked. For the most part she does so, and in doing so reaps her reward in the regard and esteem in which she is held by the medical man, the patient, and the friends.

### Nursing at the Jenny Lind Infirmary for Sick Children, Norwich.

BY A SPECIAL CORRESPONDENT.

THE new building to be opened by the Prince and Princess of Wales on Saturday owes its origin and its name to the kindly offices of Jenny Lind. In January, 1849, she was the guest of Bishop Stanley (father of the better-known Dean Stanley) at Norwich, who thus incurred the foolish censures of not a few bigots, and while she was staying at the palace the great songstress decided to give two gratuitous concerts for the benefit of the poor of the city. The amount realised was over £1,250, and the Jenny Lind Infirmary was then projected, and was eventually opened in May, 1854, in Pottergate, a quiet back street of Norwich. Jenny Lind, in 1885, wrote to the chairman of the committee: "Of all the money which God allowed me to give away, when my poor throat could call an audience to listen to its production, none has borne a nobler or more genuine fruit than the Jenny Lind Hospital of Norwich."

### THE COST OF THE UNDERTAKING.

The site for the new building to be formally opened by the "Squire of Sandringham," as Norfolk people love to call the Prince, was given by the late Mr. J. J. Colman, in memory of his wife, and Lord Leicester, Lord Lieutenant of the County, opened the subscription list for the building fund with the munificent sum of £2,000. The new hospital was inaugurated to commemorate the Queen's Diamond Jubilee, and very liberal donations and subscriptions have been forthcoming, but the cost of the building and its equipment is put at about £14,000, and an equal sum is to be raised for the endowment, of which amount a further sum of £4,000 is still required.

Part of the old Pottergate building—four large rooms, including the dispensary—is retained for out-patients; but the rest of the estate was presented to the city by the late Mr. J. J. Colman for a public pleasure ground or children's playground. At the stone-laying ceremony in connection with the new hospital on December 13th, 1898, the son and daughter of Madame Jenny Lind Goldschmidt were present, and Mr. W. O. Goldschmidt handed the trowel to Master Geoffrey Colman, who laid the foundation stone. His father, Mr. Russell Colman, chairman of the committee, with his sisters and brothers-in-law, gave £5,000 to erect the west wing, known as the Colman Ward, and devoted to medical cases; while the east wing, or surgical ward, has been built from the proceeds of a legacy from Miss Letitia Gannon, and is to be known as the "Letitia Gannon Memorial Wing."

### THE NURSING STAFF.

The old hospital indifferently accommodated 26 children; the new will accommodate 40, with every comfort and convenience that medical and sanitary science can supply, aided by the very best trained nursing. The lady superintendent, Miss L. Maude Newill, after some experience at the Nottingham Children's Hospital and a course of training at Guy's Hospital, had charge of the Hospital for Sick Children at Pendlebury before her appointment to the Jenny Lind Hospital at Norwich, and has the full confidence of the management. There are besides two sisters, three certificated staff nurses, and six probationers, also a staff nurse at Pottergate, who has charge of the outdoor patients under the directions of the medical staff. The latter all give their services gratuitously.

### THE LADY SUPERINTENDENT.

The committee of management have drawn up a code of rules with reference to the lady superintendent, *e.g.*, she must be not less than twenty-eight nor more than forty-five years of age at the time of her election, and must cease to hold office on attaining the age of sixty years. She, of course, has charge of all furniture, linen, and beds, but has to consult the lady visitor before making any special purchases. As is usual, she is held responsible for the cleanly condition of the hospital, she checks the receipt of stores, and guards against waste. Moreover, subject to the committee, she has the entire management of the nursing and housekeeping departments, with power to appoint and dismiss nurses and servants, though, in cases of dismissal, any nurse may, if she wishes, appeal to the committee, which is large and influential, ladies preponderating. The lady superintendent also keeps a register of the admission and discharge of all in-patients, is present at all operations, and attends the physicians and surgeons on their rounds. Every month she makes a detailed report to the committee of the number of deaths, if any, and of the number of children admitted and discharged. She is entitled to one month's holiday during the year.

### THE PROBATIONERS.

Probationers are required to pay an entrance fee of £5; none are received under twenty years of age, and the most suitable age is considered to be between twenty and thirty. If, after a month's trial, a probationer is thought to be in every way suitable, she will, after passing the acting physician, be received for two years' training, calculated from the date of entry. If found incapable of doing ward work, or otherwise unfit, she will not be received, and her full entrance fee will be returned, but not if she leaves of her own accord. Special probationers are received for one year's training on payment of a premium of £50, £25 of which must be paid previously to their entry to the infirmary, and £25 at the expiration of the first six months. The material for two uniform dresses is supplied by the infirmary during each year of probation. White caps, white linen aprons, turned down collars, and outside cuffs must be provided by the probationer. Laundry expenses are paid by the infirmary.

### THE NURSES' QUARTERS.

The furniture, fittings, and appliances of the Jenny Lind Hospital for Children are of the very best, and the quarters of the sisters and nurses are fitted up with everything that can be desired, good taste and comfort being combined. All the technical and scientific details have been carefully supervised by the excellent medical staff, and the position of the building, which seems to turn its back on the old city, is extremely pleasant and healthful, about a mile from the ancient boundary where the gate of St. Giles opened to the country westwards. Altogether the nursing staff will certainly work under the most favourable conditions.

### Minor Appointments.

CARDIFF WORKHOUSE HOSPITAL.—Miss Rosa Templeman has been appointed Charge Nurse. She was trained at the Wandsworth and Clapham Infirmary. She has since been staff nurse in the same institution, and charge nurse at Park Hospital, Lewisham.

EASINGTON UNION INFIRMARY.—Miss M. W. Foster has been appointed Superintendent Nurse. She was trained at the Union Infirmary, Fir Vale, Sheffield, and has since been sister at the New Union Infirmary, Wakefield. She holds the L.O.S. certificate.

HOSPITAL FOR SICK CHILDREN, NEWCASTLE-ON-TYNE.—Miss Florence Ibbotson has been appointed Sister. She was trained at Sheffield General Infirmary, and has been sister of the women and children's wards in the Blackburn and East Lancashire Infirmary.

GRIMSBY DISTRICT HOSPITAL.—Miss Amy Armitage has been appointed Night Sister. She was trained for three years at Northampton General Infirmary, and has since been charge nurse at the Scarborough Hospital and Dispensary.

### The Queen's Nurses.

### A CHAT WITH THE GENERAL SUPERINTENDENT OF QUEEN VICTORIA'S JUBILEE INSTITUTE FOR NURSES.

By Our Commissioner.

On Thursday afternoon next at three o'clock, Princess Henry of Battenberg will distribute badges and certificates to Queen's Nurses at the London Scottish Drill Hall, Buckingham Gate, when 98 new nurses will be added to the roll. In view of this interesting event I called upon Miss Peter, the general superintendent, at St. Katharine's Precincts, Gloucester Gate, Regent's Park, for the purpose of a chat about the needs and work of Queen Victoria's Jubilee Institute for Nurses.

"The institute has, I believe, now been in existence more than ten years?"

"Yes," replied Miss Peter, "the work was begun in 1889, and the first Queen's district started was in Scotland, where up to that time nursing the poor in their own homes had never been attempted. In England the movement had commenced earlier. It owed its initiation to Miss Florence Nightingale, and it was first put into practice in Liverpool in 1859. In 1876 similar work was begun in London, and the Metropolitan and National Nursing Association was formed. But the Queen Victoria Jubilee Institute—now recognised as the head centre of district nursing in Great Britain and Ireland—did not start till thirteen years later."

### The Number of Nurses.

"How many nurses are now employed?"

"Roughly speaking, about one thousand, sixty of whom are Welsh speaking nurses, who work in Wales only."

"Does the supply exceed the demand?"

"Not at all. We could find work for many more nurses if we had them, and at the present time especially, owing to the unrest produced everywhere by the war, we find it particularly difficult to cope with the requests for nurses for district work. And yet the work has many advantages."

"What are the qualifications necessary to become a Queen's nurse?"

"All candidates must have received not less than two years' training in a good general hospital. Many who come to us have had three years and longer, but we accept probationers with two years only."

### The Question of Training.

"Unless a nurse has already been trained it is, of course, useless for her to try to become a Queen's nurse?"

"Oh! yes; because if a candidate wishes we can arrange for her to have the necessary hospital training, though in that case the period of engagement to the institute is necessarily longer."

"Where are your training hospitals?"

"They are scattered all over the country for the convenience of probationers. Amongst others I may mention the London Hospital, the Edinburgh Royal Infirmary, the principal training schools in Dublin, Cardiff, Chester, Shrewsbury, Worcester, &c."

"Is any salary given during the training?"

"Yes, a small salary; and all fees, of course, are paid."

"Then as to special training in district work."

"That is given, after a month's trial to prove the suitability of the candidate, in the training homes affiliated to the parent institution. The nurses remain for six months, and during that time they not only have practical work in the districts attached, but they also have three free courses of lectures touching on subjects a special knowledge of which will be most useful to them in district nursing, more particularly the diseases of women, and invalid cookery. Midwifery training at the expense of the association is likewise given where it is thought desirable."

"Is nothing further necessary to become a Queen's nurse?"

"The nurse must enter into an agreement to remain with us for one or two years after the completion of her training, and her name must have been submitted to the Queen and approved of."

### The Title not Nominal.

"Then the title 'Queen's Nurse' is something more than a name?"

"Certainly; twice a year, in January and July, the list of the nurses who have completed their training and are ready to be enrolled, together with particulars of their age, religious denomination, hospital and district training, is sent to Windsor, and returned endorsed by Her Majesty."

"Perhaps," continued the Superintendent, "you might like to see the list," and she handed me the folio. There in the top left-hand corner was the well-known signature, "Victoria R.I.," written strongly and firmly beneath the one word, "approved," also in the Queen's handwriting. "Until the list has been returned the nurses do not receive the notification that they have been appointed Queen's nurse, nor are they given their badges."

### Special Advantages.

"You spoke earlier of special advantages in becoming a Queen's nurse. To what did you allude?"

"To many the independence of the work is specially attractive," replied Miss Peter. "No nurse is allowed to undertake nursing in a district for a local committee unless she is provided with two furnished rooms, with attendance, fire, and light; with 12s. a week allowance for board, and laundry paid to the nurse herself; £4 a year for uniform, and £30 per annum for salary, rising to £35 the third year. Thus the nurse has, as it were, her own little home instead of living in an institution. When necessary, we allow this home, by special arrangement, to be shared by a mother or sister, so that some women, who would otherwise be tied to home duties, are able to do good work. Besides the ordinary Queen's nurses, there are the inspectors and superintendents, whose salaries vary from £60 to £100 a year, and the greater the number of homes affiliated the more numerous become these posts."

### Pensions.

"Have you any pension or sick fund?"

"Sir Henry Tate presented us with £5,000, the interest of which goes to help sick nurses. Twenty-two were benefited by the fund this year. As yet, however, we have not been able to fulfil our great wish to assist our nurses to subscribe to the Royal National Pension Fund, with which we are much in sympathy. This is simply from lack of funds, and we hope to be able to make some arrangement later on."

"Finally, how many of your nurses joined the Army Nursing Service?"

"Eleven Queen's Jubilee nurses are at present in South Africa, but we hope that they will return to us when peace is declared. Most of our nurses come back to us when they can. Even if they leave for private work, or for other reasons, they frequently join us again, because they say they have never elsewhere been so happy and so independent."

And then, having admired the box full of badges and brassards tied up ready for Princess Beatrice to present next week, I took my leave.

### The "Nursing" of the Sick Soldiers in South Africa.

### SERIOUS INDICTMENT AGAINST THE WAR OFFICE.

In the *Times* of Wednesday there is a letter from Mr. Burdett-Coutts, M.P., under the heading "Our Wars and Our Wounded," which, as our contemporary says, is neither more nor less than a serious indictment against the War Office. Mr. Burdett-Coutts admits that the arrangements for tending the wounded at the beginning of the war were fairly satisfactory. It was not, he goes on to show, until pressure came from the fearful increase of typhoid cases that the nursing arrangements proved so disastrously inadequate.

### Ten Thousand Typhoid Patients.

There are, Mr. Burdett-Coutts estimates, 20,000 sick and wounded troops in South Africa, and more than half of these are down with typhoid. The date of his letter, written from Cape Town, is May 29. He thus describes the way in which the sick are nursed, or not nursed, at Bloemfontein: "An ordinary field hospital contains 100 beds—a *façon de parler*, because it has no beds. When stretchers are available they are sometimes used as beds in the tents. The theory of a field hospital is that it is to be always moving with troops. When troops make a long stay in one place it may be used as a 'stationary' hospital, and its equipment should be improved for that purpose. It is an axiom laid down by an accepted authority that 'if the hospital is to be long in occupation every effort should be made to raise the patients off the ground.' Ten weeks' existence of this hospital in one spot is certainly a period satisfying the condition; but no attempt was ever made to supply beds or even mattresses for it. Situated within a mile of Bloemfontein, nurses could have been accommodated in it just as well as in a 'general' or 'stationary' hospital, where they are allowed. There were never any nurses in it, or, indeed, in any field hospital. The distinction between a field hospital used as this was and a stationary or general hospital is rendered merely nominal by strategic security, permanence *in situ*, and enlarged accommodation. In these respects some of the field hospitals around Bloemfontein differed in no way from general hospitals, and least of all in the necessity for proper nursing. But hardly any nurses at all came to Bloemfontein for a month after our occupation of it."

### Ten in a Tent and No Nurse.

Later on Mr. Burdett-Coutts paid a visit and saw for himself the following state of things: "With no further equipment than two marquees and a few bell tents, no addition of staff or anything else, there were 316 patients, of whom half were typhoids. Their condition was almost indescribable. The tents were bell tents, such as were mentioned in a former letter as affording sleeping accommodation for from six to eight orderlies when working and in sound health. In many of these tents there were ten typhoid cases lying closely packed together, the dying against the convalescent, the man in his 'crisis' pressed against the man hastening to it. There was not room to step between them. Think of this, you who know the sort of nursing a typhoid patient requires. With no beds or mattresses, and only 42 stretchers in the whole hospital, it followed that 274 patients had to be on the earth. There was a great scarcity of blankets, and no patient could have more than one, with a waterproof sheet between his body and the ground. The ground is hard as stone, and at night the temperature falls to freezing point. Besides other deficiencies which cannot be described there were no sheets or pillow-cases or pretence of bed linen of any kind; only the coarse rug grated against the sensitive skin burning with fever. The heat of these tents in the midday sun was overpowering, their odours sickening. Men lay with their faces covered with flies in black clusters, too weak to raise a hand to brush them off, trying in vain to dislodge them by painful twitching of the features. There was no one to do it for them. Seventeen orderlies had come with, or been raised for, the half-section of the field hospital; ten had been taken from it, the number being made up from the Bearer Company, but they had other duties to perform than brushing flies off patients' faces. At night there were not enough to prevent those in the delirious stage from getting up and wandering about the camp half naked in the bitter cold. In one tent, where some slept and others lay with eyes open and staring, a case of 'perforation' was groaning out his life huddled against his neighbour on the ground. Men had not only to see, but often to feel, others die."

### A Sad and Sickening Spectacle.

It was a sad and sickening spectacle this, which I describe exactly as my eyes saw it, and without exaggeration or excuse. I leave it and other similar facts it will be necessary to relate to the consideration, not of wives and mothers—we will put them out of sight—but of hard, practical men, accustomed to the hospitals of the poor, of the medical profession, of the great nursing community, of the whole British public, who at the moment when this sight was to be seen out here were reading those comforting words spoken at Calais on April 26th, as an avant-courier of the speeches two days later at the Reform Club: "Nothing that prevision could suggest or that money could purchase was wanting anywhere. The supply was simply lavish. . . . Here everything was sent up with the utmost promptitude, and medical stores and comforts were always on the spot."

### No Fault of the Medical Officer.

As to the question of local responsibility, Mr. Burdett-Coutts affirms that "certainly in the case of this particular field hospital it did not lie with its chief medical officer, who was an energetic, painstaking member of the R.A.M.C., working day and night, never leaving his hospital, and sitting down each evening to his blue paper 'returns' after 14 hours' work in the tents. 'Yes,' he said simply, as we parted, 'we do our best, but it makes one's heart sick to look at them.'"

### 520 Patients and No Nurses.

Mr. Burdett-Coutts continues: "There is no need to pile on the agony, but worse remains behind which must be told. Therefore I will pass over an incidental visit to the hospital after a heavy rain, when many of the patients—typhoid had increased—were to be seen lying three inches deep in mud, and come to my last visit, four days ago, on my way down to the front. The chief medical officer had been changed; from all reports this one was as painstaking as the last. He told me that at one time his patients had increased to 496! Three hundred of these were typhoids. The few trained orderlies had been mostly taken away; in their place were 25 untrained and ignorant privates from an infantry regiment, most of whom were themselves 'convalescents,' to do the whole of the nursing. The medical staff remained always at three. The patients here were within 24 of the number (520) allotted to a general hospital. A general hospital has 20 medical men, 78 trained nursing orderlies, 27 untrained privates, and nine nurses. The sick require far more attention and nursing than the wounded; the general hospitals at Cape Town, equipped as above, were mostly occupied by wounded. Here was this hospital crowded with typhoid left to three doctors, 25 untrained privates, and no nurses."

### The Royal British Nurses' Association.

ANNUAL MEETING.

THE annual meeting of the Royal British Nurses' Association was held on Saturday afternoon in the Imperial Institute. The room lent for the purpose was the Australian Chamber, and Sir JAMES CRICHTON-BROWNE, who was in the chair, remarked that the name was particularly appropriate, seeing that the meeting was asked to sanction the formation of a South Australian branch.

Sir C. G. BROWN moved, and Miss GEORGINA SCOTT seconded, the resolution dealing with the first Colonial branch of the Association. The former described the birth of the Royal British Nurses' Association (at which he and one other hon. officer present, Miss Thorold, had attended) in medical terms; it was a healthy infant, and a pleasant child; it showed no evidence of heredity (so much discussed nowadays), and no indication of delicacy. "But," he went on, "it had to suffer the diseases of children, and it had especially a very bad attack of malaria. Nevertheless, owing to its excellent nurses—both male and female—it pulled through; though if there hadn't been a Royal nurse in it, it would have died. To-day it is grown up—as children are at twelve years old in the east, though not in the west—and we are called in to assist at the birth of a child, the starting of a new branch in the south. I believe that with such a destination and in such a climate, and with the wife of the Governor of South Australia (Lady Tennyson) as vice-president, it has an assured future before it. But it is to be kept a child, and guided by rules, though it will be a twentieth century child, with as much freedom and liberty as it is possible to give it consistently with loyalty."

In seconding the resolution, Miss SCOTT said that the starting of the new branch proved not only that the colonies needed us, but that we needed the colonies. She had just been reading the report of the Adelaide Hospital, and thought that in every way it compared favourably with our metropolitan and provincial hospitals.

THE ANNUAL REPORT.

The HON. SECRETARY (Mr. Fardon), in moving the adoption of the annual report, said it was desirable that those engaged in nursing in the colonies should adopt the methods of organisation and secure the same training as in English hospitals, and that this desire was gradually permeating those in the colonies. A large number of nurses had already been registered.

AN AUXILIARY SOCIETY.

"It is interesting," says the report, "to learn that material progress has been made in the formation of an auxiliary society whose object is to extend to many highly-trained members of our Association, who by reason of their age are unable to join the Chartered Nurses' Society, opportunities of employment on co-operative lines. There is every probability that this new society will be able to commence work in the early autumn." It was not the province of the Association to find work for its members, the Hon. Secretary said, but it should seek by all means in its power to promote any other movement for doing so. Therefore it was with some pleasure that he referred to the Society of Chartered Nurses, which, although not managed by the Association, had on its committee many interested in it. The number of members was not very large, but it was increasing every year, and there was every prospect that the Chartered Nurses' Society would be the means of finding employment for a very large proportion of members of the Association.

The object of the Auxiliary Society of Nurses was, he continued, a most legitimate one. The Chartered Society had restricted its membership within certain limits of age; but it appeared to many members of this Association that a large number of nurses with wide experience and good capabilities were thus left out. They had therefore determined to found an auxiliary, which would not interfere with the Chartered Society, but which would offer work on co operative principles, and which, it was believed, would provide the means of remunerative employment to many members of the Association.

Miss HUTCHINSON also thought that the Auxiliary Society would fill a gap, and be a boon to many nurses.

"Those of us," she said, "who have thought out the subject, must have been much exercised in mind as to what was to become of nurses after 36—the age limit of the Chartered Society. It is iniquitous that women who do not begin their training till the age of 23 or 24, and who then have to spend three years upon it, should be put on the shelf at 36—just when they are beginning to profit by their knowledge and to see how much there is still to learn. The Association is helping to solve the problem."

HOW THE HOSPITALS HELPED IN THE WAR.

Miss WEDGEWOOD, matron of the Royal Free Hospital, in seconding the adoption of the report, said that all the hospitals had responded nobly to the call for nurses to go to South Africa. The largest number had been sent by St. Bartholomew's. A few weeks ago she had mentioned the fact on a Monday that nurses were needed at the Yeomanry Hospital, and by Saturday several were on their way to the Cape. St. Thomas's had also sent nurses to the Yeomanry Hospital, which was worked by the Army Nursing Reserve, but separately. St. Mary's, the Middlesex, and Guy's had also sent nurses. The last had given its best sister, and she had ever since been in the Hospital Train, which had met with no accident all through the war. Mrs. Strong, of Glasgow Infirmary, was supplying a large body of nurses, who would sail on June 30th, and these would bring the number of the reserve up to 490.

DEATH IN THE RANKS.

The deaths, Miss WEDGEWOOD observed, had been particularly sad, and she mentioned especially Miss Florence Bell, who was so bright when she left for South Africa, but whose health gave way, through weakness of the heart, in one week. She was at Modder River visiting the nurse in charge of No. 2 train not long before her death, and laughingly remarked that she had always thought there was no work to do on a train, but had changed her opinion after seeing the process of "sponging the Tommies."

Three other members of the Association have died during the discharge of their duties on the Army Nursing Reserve in South Africa, and they were spoken of by name by the hon. secretary. Mrs. Clayton, better known as Sister Collins, had worked at Cheltenham and Shrewsbury; she was at Kimberley through the siege in charge of the Christian Brothers' Hospital. She went to Kimberley in response to Lord Roberts' call for more nurses, and succumbed to enteric fever. Miss Florence Sage was well-known and greatly beloved at Taunton; she died at Spitfontein on June 12th. Miss Bonella Hall, who was sister over an important ward in the Middlesex Hospital, was also a victim to enteric fever.

THE SETTLEMENT.

The formation of a home for aged nurses, already discussed by the general council of the association, was alluded to by several speakers. The hon. treasurer, Mr. LANGTON, spoke of it in moving the adoption of the balance-sheet, and so did Miss HUTCHINSON in supporting the annual report. Nearly £150 has already been subscribed for the purpose. The fund has received the direct sanction of Her Royal Highness, the President of the Association, and many influential members of the general council, together with the lady consuls, are lending their cordial co-operation in order to bring it to a successful issue.

A comfortable bed, Miss Hutchinson thought, was one most important point in furnishing the proposed settlement. She would like to suggest the most comfortable that could be got. Sometimes it happened that in nursing private cases nothing was available for the nurse but the common or garden chair bed, though as a nurse she spoke most gratefully of the kindness of her patients. It would be a great comfort to a woman who had worked hard, and borne the burden and heat of the day, to have a comfortable bed on which to rest in her old age.

"If," added Miss Hutchinson, "I thought that the out come of this meeting would be £100, a sum which would, I believe, provide one room for one nurse, I would gladly give one guinea if 99 others would do the same."

The following extracts are from a slight sketch of the settlement which was distributed throughout the room. The words are those of Miss Hutchinson, when speaking on the subject at the general council meeting:—

"The size of the settlement must, of course, depend on the amount of money which is forthcoming to build it. It has been estimated that quarters for ten nurses could be built for £1,200, and that as many as twenty nurses could be comfortably accommodated at a cost of £2,000. All these nurses must be in possession of a small income of at least 8s. or 10s. a week, for it is not proposed to attach any money allowance to the free quarters assigned to them. . . . It is not proposed to build the settlement in the form of separate small cottages, but it has been proposed that each nurse's quarters should consist of one good room, and that these rooms should open on corridors warmed and lighted in winter, with bath-room and lavatory on each floor. The rooms will be fitted up with the most careful eye to the convenience of the occupant, and it is hoped that by the use of various modern appliances there will be a great saving of labour and avoidance of discomfort. The occupant of each room will be its undisputed mistress. . . . An essential feature of the settlement will be a common sitting-room or club-room, provided with books, papers, and perhaps a piano, where the nurses can meet each other and their friends, and where they may find, if they desire it, a little relief from the monotony of their own room. In summer a few flower-beds and a grass-plot, where they may sit under shady trees, complete the picture."

Dr. Bezly Thorne moved a vote of thanks to the hon. sec. and other officers, and Miss Katherine Scott seconded. In the course of his remarks the former alluded to the part taken by Lord Sandhurst in the formation of the Army Nursing Reserve, the idea of which some placed to the credit of himself, and others to Her Royal Highness the Princess Christian. Lord Sandhurst, at that time Under Secretary for War, had given it a great impetus in urging him (Dr. Bezly Thorne) to bring it before the notice of the Princess.

A vote of thanks to Princess Christian was proposed by Mr. Pick, and seconded by Miss Thorold.

The member of the Association who was to have moved the resolution about uniform was not able to be present, and the matter was referred to the next General Council.

Other resolutions concerned the election of the general council, two new vice-presidents (Sir C. G. Brown and Lady Tennyson), the election of the auditor, and thanks to Sir James Crichton-Browne for taking the chair, and to the Fellows of the Imperial Institute for lending the room in which the meeting was held.

### Presentations.

Ipswich Nurses' Home.—Miss Garrett, who is resigning her position as lady superintendent of the Ipswich Nurses' Home, has been presented by the nursing staff with a very handsome dressing-case, containing a full set of silver-mounted bottles, brushes, &c. They were enclosed in a Russia leather case, with a silver plate inscribed: "Presented to Miss Garrett by the nursing staff of the Ipswich Nurses' Home, May, 1900." The cottage nurses availed themselves of the opportunity by joining with the nursing staff to present Miss Garrett with a complimentary address engrossed on parchment, worded as follows: "Presented to Miss Garrett, lady superintendent, by the trained nurses and cottage nurses of the Ipswich Nurses' Home on her resignation, and in token of their appreciation and the high esteem in which she was held by them all. Thanking her for the kind interest she has taken in them, wishing her every happiness and success in her future life." To this address were attached forty signatures. Miss Garrett, in acknowledging the gifts, heartily thanked the nursing staff for their kind feeling towards her, and said she could only repeat what she had remarked on many occasions, "That the Nurses' Home had every reason to be proud of the efficiency and good tone of the present nursing staff." During the four years and nine months Miss Garrett has been at Ipswich she has much improved the private nursing staff, and started and successfully organised the present up-to-date district nursing—a system which she found on entering on her work for the Nurses' Home was greatly needed by the sick poor of Ipswich. In the last nine months 421 patients have been attended, and over 12,000 visits paid by the district nurses under Miss Garrett's superintendence.

Whitby Cottage Hospital.—Miss Julia Mercier, who has been appointed matron of the Alton Cottage Hospital, and Miss L. M. Button, who accompanies her as assistant nurse, were presented before their departure from Whitby with handsome and useful testimonials. To Miss Mercier was given a silver tea set, Queen Anne pattern, with the following inscription engraved on the teapot: "Presented to Miss Mercier by many friends in Whitby in grateful remembrance of three years' work amongst them in connection with the District Nursing Association and the Cottage Hospital. May, 1900." Miss Button was the recipient of a handsome travelling clock inscribed with her name and date of presentation.

Cheyne Hospital for Children, Chelsea.—Last week a testimonial, consisting of a purse containing £83, and an address, illuminated on vellum by Miss Lilian Holt, were presented to Nurse Jane Teague, to commemorate the completion by her of twenty-five years of faithful and devoted service.

### To Nurses.

We invite contributions from any of our readers, and shall be glad to pay for "Notes on News from the Nursing World," or for articles describing nursing experiences, or dealing with any nursing question from an original point of view. The minimum payment for contributions is 5s., but we welcome interesting contributions of a column, or a page, in length. It may be added that notices of entertainments, presentations, and deaths are not paid for, but, of course, we are always glad to receive them. All rejected manuscripts are returned in due course, and all payments for manuscripts used are made as early as possible at the beginning of each quarter.

### Nursing in America.

By an English Nurse.

III.

In the second week in January I returned to find Boston transformed. It had become a white, fairy-like city, lying under a dome of the most wonderful transparent blue, and glittering under the brightest of sunshine—a city in whose streets all noise and clamour of traffic had given place to the jingle of sleigh bells. Having reported myself at the "Directory" as ready for work, and finding that there were some hundred and odd nurses' names on the list before mine, and that the chance of being "wanted," at least for a time, was small, I set about enjoying myself as well as I could.

### The Social Position of American Nurses.

What with one thing and another, I occupied a fortnight comfortably enough, and then I began to get tired, desperately tired, of the waiting and the idleness. I was not used to idleness, I had always been in the position of having more work than I could do, and I had never been good at "waiting." During that time I saw more of my fellow-nurses than I had hitherto done, and was able to study them better. I found out that the difference between them and others I had met was not so wide as I imagined. The type was in some cases the same, the difference being more in the want of breadth of outlook, the narrowness of view. This I could only wonder at for a time, till, by degrees, I found out that while in some respects, and professionally, the American nurse is placed on a higher plane than her English sister, in another, that is, socially, she ranks much lower, and that her life is generally a lonely one, and her intercourse limited practically to women of her own profession. As soon as she has graduated and taken her diploma, the American nurse stands in pretty much the same position as regards independence that a doctor would here; but as regards social standing, "Why, anyone would as soon ask their cook to lunch or dine with them as a trained nurse," my room-mate remarked, with some bitterness. Slowly another week, and then another, dragged on. I enjoyed the lovely clear air, the brilliant sunshine, and astonished everybody with the length of my walks and variety of my exploring expeditions, but I paid few calls, and refused all invitations. My conversation with the other nurses had given me an uncomfortable feeling that I was in a false position; that if that was how nurses were regarded in America generally I had probably caused the people who had extended their hospitality to me so generously as "a friend from England," and had remained so loyal in their friendship—in spite of the fact that I was but a working woman, working in their midst—some uncomfortable moments in introducing me to their friends. Expenses were mounting up, too; of course, the rent of my room went on whether I occupied it or not, and Boston is not the place to live on air. Personally, it had endowed me with a first-class appetite. Certainly, meals at cafés, as compared with our standard, were cheap. But, even living economically, money went, and stamps, car fares, &c., mounted up; and, as everyone united in saying it was the healthiest, and, from doctors and nurses' point of view, the dullest, season ever known in Boston, I began to wonder if I had not better move further afield.

### An Opening.

Indeed, my wisest plan, especially as I had accomplished my purpose of having a winter in America, seemed to be to return home, and I had begun to make inquiries about sailings and berths, when the opportunity of entering a small hospital of 50 beds up in the mountains, where I had heard indirectly that surgery was exceptionally good and up-to-date, was offered me. The agreement was that I was to take night duty, but was to be allowed to attend and assist at all operations. I was to pay my own expenses there and back—which, as the distance was considerable, came to about £4—and I was to receive £6 per month, which is about the rate of remuneration for a good domestic servant in the States.

### The Business of Nursing.

The nurses in the house strove to dissuade me from going "It is absurd," one observed, "you, a graduate, with so much experience, going back to that drudgery." I mildly suggested that my hospital experience had hitherto consisted of hard work, but happy work, and we never thought of counting our hospital days as drudgery. "Well, ours are, and we put up with it simply because we must if we want to make a living at nursing; but you will seldom, if ever, find an American fool enough to remain in hospital a day after she has graduated." And that sentence explains the situation and status of the average American nurse. She takes to nursing as she would take to any other business or profession, simply as a means of making money. Of course, there are exceptions, and it was my good fortune to meet more than one. My friends looked somewhat doubtful when I went to say good-bye the day before starting, and it was not with any very great cheerfulness that I set out for my destination next morning. "One thing, it is sure to be a good hospital, for it is in the capital of the State, the only one, I believe, for a hundred miles round, and it is very beautiful among the mountains. You will be able, too, to see what a New England winter is really like up there. You must not judge them by Boston this winter," one of the family had said cheeringly the day before; so bearing that in mind my spirits rose.

### My First Night at "Claremont."

As the day wore on we climbed steadily higher and higher, leaving the snowless country round Boston and Concord behind us until we got to the region of the snow, and from every wayside station we passed the icicles hung in clusters a yard long. It was evening when I got to the end of the journey. Everywhere snow lay, and when presently we emerged from the city—such a queer little city as it seemed driving through it that first night, with its shabby little shops and its glaring electric light—and dashed into the open country round the hospital, the runners sank into snow that looked at least two feet deep. I am not likely to forget my first night at Claremont. I had been travelling all day, and at ten o'clock I had to go on with twenty-five strange patients—most of them paying. They were scattered up and down—in single private rooms, in wards holding two, three, and four. They were of all kinds and classes, and were suffering from all sorts of diseases. There were two amputations—one of both feet—done that day; there was a case of acute rheumatic fever, and a case of removal of rectum from cancer, operated upon two days before. A case of senile decay, and a slave to the morphia habit, occupied rooms on each side of a young mother and her child, and every one of the twenty-five patients possessed an electric bell. I wonder my hair did not turn grey that night over those bells. Most of the small wards had names, but it took me some time to remember that the third without a name meant the cancer case; and the one marked "head nurse," the girl with rheumatic fever; that surgical ward meant one where three women slept; and main ward that occupied by four men. The duties of a night nurse are pretty much the same everywhere; mine began at seven p.m., when I started administering the last dose of the three times a day, and evening medicines and nourishments till seven a.m., when, having washed, or seen washed, everybody all round, and given them all their early coffees, I sat down to my own breakfast.

### Echoes from the Outside World.

### AN OPEN LETTER TO A HOSPITAL NURSE.

The news which reached us on Monday of the defeat of the column of Russians and Americans who had gone to the relief of Tientsin, though a matter of much regret, occasioned no surprise, more especially as it is said that the Chinese are very good soldiers, who fight well, when they are armed, as in the present instance, with modern guns and up-to-date field pieces, and are also inspired with fanatical courage. Therefore the news which came on Wednesday was doubly welcome. It appears that the little relief force, though only consisting of 3,000 men, had really succeeded in breaking through the cordon of 20,000 Chinese, and had entered Tientsin on Saturday night, with but trifling loss. Evidently the rumour that the town had been utterly destroyed was as false as that which described the allied forces as cut up, and that alone is a matter for great congratulation, as it is a very large city, twice as big as Manchester, and the result of nearly fifty years' unceasing activity on the part of British and other enterprise. The relief of Tientsin has naturally made us hopeful that satisfactory information with regard to the safety of Admiral Seymour and Pekin will follow shortly. But it is strange how history repeats itself. When the Boer war came we were all unprepared, whilst our enemy had been laying in martial stores for a long time. Now, again, in China we have only a handful of men available, whilst according to a telegram from Shanghai, the Chinese have been quietly accumulating rifles for the past three years at the rate of 20,000 a month.

---

We were told so much at the beginning of the South African war of the antagonistic sentiments with which we were regarded by the United States, and the delight which the Americans experienced at the reverses which befel us, that notwithstanding the pleasant incident of the "Maine"—by the way it may interest you to know that Lady Randolph Churchill is going to be married again to Mr. Cornwallis-West—that we had almost commenced to believe that the majority of the folks in the land of Stars and Stripes disliked us. At any rate, it was refreshing to hear the other evening from the Bishop of Washington an emphatic denial of these assertions. He stated, with some warmth, that with the exception of a few organs of the Press, and a very small minority who are pro-Boer in their sympathies, the people in his important diocese are unanimous in their goodwill to England. The rejoicings over the relief of the besieged cities, and the entrance of our troops into Pretoria, had, he said, been almost as great in America as it had been in some parts of England; and Dr. Satterlee also spoke very strongly of the different way he had been received here now and upon the occasion of his last visit. Then, John Bull had been somewhat on the defensive. He was by no means sure that Brother Jonathan was perfectly friendly, and he noticed that an undercurrent of not altogether pleasant feeling intruded itself into the intercourse. This year, however, he finds that a very different state of things exists. Everywhere the hand of goodfellowship is extended to him, and he is made truly welcome. This is, he declared, what it should be, "because England and America are the only two countries which thoroughly understand the meaning of Constitutional Liberty."

---

Nurses who reside in London and take delight in art have another pleasure added to their lives. On Monday there was opened to the public one of the most marvellous collections of paintings, armour, bronzes, enamels, china, and furniture which has ever been got together. I allude to the Wallace collection at Hertford House, Manchester Square. It was begun by the third Marquis of Hertford, and carried on by his successor, Richard, the fourth Marquis, who resided for many years in Paris, and who upon his death left all his wonderful works of art, and Hertford House itself, to Richard Wallace, his companion and friend, and as some believe, his near relative. This was in 1870, and therefore additional interest is lent to some of the treasures from the fact that they passed unhurt through all the horrors of the Franco-German war. But their owner, recognising that such a disordered capital was no fitting home for priceless gems, moved them to England in 1872, and it may be remembered that they were exhibited at the Bethnal Green Museum for three years, whilst Hertford House was being made ready to receive them. They have remained in Manchester Square ever since. At Sir Richard's death, soon after he had lost his only son, he left everything to his wife, and she, in her turn, bequeathed all to the British nation, on the condition that the collection should have a special home assigned to it in Central London. This has been accomplished by purchasing for their accommodation their original resting place, Hertford House. I cannot tell you of half the beauties you will find in the galleries. The most notable, perhaps, are the examples of the French school of painting, which are so numerous and so comprehensive that they entirely take away the reproach, which was at one time well deserved, that we had wholly neglected the French school in our public collections in England. The Watteaus, in particular, nine in number, are lovely. "The Champs Elysées," which, though small, is in splendid condition, and "Les Amusements Champêtres" are noteworthy, and should be specially valued by us, because the National Gallery, strangely enough, has not a single example of this charming painter. The Greuzes, too, are so fine that they are said to be the best collection in the world, and are most fascinating in their dainty beauty. I must not write more just now, except to advise you to seize the first "off duty time" you can for a visit to Hertford House—the exhibition is open from 10 to 6—but next week I hope to return to this subject.

---

I went to see "Rip Van Winkle" at Her Majesty's Theatre a few days ago, and was delighted with it. Mr. Tree is an ideal Rip, and despite the feeling of admiration that one could not help having for the beautiful Gretchen as portrayed by Miss Hanbury, whenever Rip appeared on the stage the fascination of the character immediately alienated one's sympathies from the wife. Rip's return to the Town of Falling Waters after, as he supposed, his one night's stay on the mountains, which was in reality a twenty years' sleep, is most pathetically rendered. The charm of the whole production is greatly enhanced by the scenery. Her Majesty's Theatre under present management is synonymous with beautiful productions, and the present version of "Rip Van Winkle" easily sustains this reputation. The ravine in the Kaatskill Mountains and Sleepy Hollow are especially effective, each in an entirely different way. Altogether "Rip Van Winkle" is a play which should not be missed by those who want to enjoy to the utmost an afternoon or evening at the theatre.

### A "Nurses' Settlement" in New York.

ON Friday last the warden, Miss Honnor Morten, and members of the Hoxton Settlement entertained three American nurses, Miss Wald, Miss McDowell, and Miss Waters, of the Nurses' Settlement, New York, to tea at Essex Hall, Essex Street, and invited a few friends to meet them and hear something of their work.

Women's Settlements have sprung up in plenty during late years in the crowded and poorest parts of London, but none of these are founded quite on the lines of the New York experiment. Miss Wald's brief account of how the scheme originated "with two nurses," one of whom it is safe to assume was herself, greatly interested her audience. The sight of the teeming wretchedness of the most densely populated and squalid part of New York, practically the "foreign quarter," where nationalities of all kinds gather and herd together in tenement houses, inspired these nurses to go themselves to live amongst these people, and to do what they could to better the social conditions they found around them. Seven years ago they started their work on the top floor of a tenement house in this region of New York, and as time went on they gathered to themselves more helpers, and became the centre of a busy little community. Now, there are some twenty nurses living together in a small house, working, so far as the nursing, their real *raison d'être*, is concerned, more or less on the lines of the Queen's Jubilee Nurses, closely in touch with all the hospitals and with the principal medical men in the city, yet in a free and untrammelled way which has greatly helped their development as a social force.

Gradually, the "Nurses' Settlement" became a power in the land; the Board of Health supported the work, and they were able to draw the attention of the authorities to abuses of the sanitary laws, and so to secure urgently-needed reforms. Besides the purely nursing part of the scheme, other developments came about by degrees. Additional helpers, not trained nurses, joined the Settlement as associates, non-resident, who aid in the management of boys' clubs, kindergarten, and other affiliated organisations; a house on the Hudson River for the over-worked and over-tired, and a camp for convalescents, &c., have been added to their schemes, and all have worked well.

The latest development has been in connection with the nursing. Recently, the New York training schools, in common with those in other parts of the States, have added a third year to their course. It was suggested to the authorities that nurses who had completed their two years' might be sent to the Settlement for three months' experience in district work, an invitation which has been accepted. The training thus provided will be of great value to the graduate nurses of the New York hospitals. Thus, out of small beginnings Miss Wald's energy and enthusiasm have evolved an organisation which must be a powerful agent for good in its influence on the social life of the poor in New York. The training of a nurse specially fits a woman for dealing with the miseries and sanitary evils which result from overcrowding. She is welcomed where the inspector is shunned, and it is in her power to do more to educate and alleviate than is the case with many other reformers. The success of the Nurses' Settlement must greatly encourage its members in their good work.

### Novelties for Nurses.

BARGAINS IN LINEN.—Messrs. Robinson and Cleaver commenced their summer sale at their establishment in Regent Street on June 25th. Their catalogue shows that an excellent opportunity will be afforded purchasers to secure good materials or articles of wearing apparel and household use at wonderfully moderate prices.

### Everybody's Opinion.

**[Correspondence on all subjects is invited, but we cannot in any way be responsible for the opinions expressed by our correspondents. No communication can be entertained if the name and address of the correspondent is not given, as a guarantee of good faith but not necessarily for publication, or unless one side of the paper only is written on.]**

### ROMAN CATHOLIC NURSES AND GUY'S HOSPITAL.

The HON. SYDNEY HOLLAND, Stonewall Park, Edenbridge, writes: "A. F. C.," writing in your issue of the 23rd, under the above heading, says Roman Catholics contribute as largely to hospitals as members of any other Church. "A. F. C." is wrong. There is no body, no creed, who contribute so little, in proportion to their numbers. I have before now written to Cardinal Vaughan pointing this out, and the reply I have received is that the Roman Catholics have to contribute so much to their own special charities. I have not the figures by me at the moment, but I shall be surprised if it be found that more than £500 was collected last Hospital Sunday amongst all the Roman Catholic places of worship. Why, double that sum has often been collected in one place of worship of the Church of England, and at least £500 might reasonably be expected to be collected in the Oratory alone. And everyone of us has his own special charities to help in addition to hospitals. I think that the fact how very little Roman Catholics, as a body, give to hospitals should be known, and perhaps they will amend.

### NURSES' READING SOCIETY.

MISS MOBERLY writes: I am glad to be able to report at the end of the first year of this society that it is in a most flourishing condition. We now have sixty-eight members. Those nurses who have answered the examination questions set quarterly have shown that the books set have been thoroughly and conscientiously studied, and the marks gained have been high on an average. By the beginning of October (after the current quarter's examination questions have been sent in) I shall be able to announce the names of the two nurses who have gained the prizes for the year. A kind friend has given a small sum of money which will be divided between the two nurses who have made most marks in the four quarters. The books read during the year that began last July have been "Tuberculosis;" "Mental Nursing," Harding; "Hygiene," Glaister; "Surgical Wardwork," Miles; and "Children's Nursing," C. J. Wood. For the quarters beginning in October and January next I propose setting "A Manual of Obstetric Nursing," by M. Humfrey, taking one volume for one quarter, and the second volume for the next quarter. I hope this arrangement will meet the wishes of the Nurses' Reading Society. It seems to me that the subject is such an important one that a careful study of it will be valuable to every nurse.

### OUR DUTY TO THE DYING.

"NURSE H." writes: I have often wondered as I have stood by the bedside of the dying whether my patients would like to know the end was near, and it has saddened me many a time to see how careless they are as far as the soul is concerned. "Would you like a chapter read to you from the Bible?" I have often asked, "St. John is so comforting." "Oh, no, nurse, I thank you," has been the answer, "I will wait until to-morrow." In a few instances to-morrow never came. We do meet with cases like that sometimes. Of course it is a mistake to force religion down anyone's throat. We also meet with others like "Patient," who wish to be told because they have so much to do before the end comes—loving messages to send, perhaps to those who are thousands of miles away. I think one of the most beautiful death-beds I have stood beside was that of a beautiful golden-haired boy. It was just twelve years ago. He was only five and a half years old, he was truthful, and so sweet. The dear little fellow knew he could not get better, and often when he had scarcely strength to speak he would say, "Nurse, will you please sing to me?" I used to comply with his wishes and try to sing softly all the beautiful hymns he selected, until he would say, "I am so tired, don't sing any more to day."

### For Reading to the Sick.

"Humble yourselves therefore under the mighty hand of God, that He may exalt you in due time; casting all your care upon Him, for He careth for you."—1 *St. Peter* v. 6, 7.

And is the Hand that formed now laid on thee
In chastening—Fatherlike? Art thou distrest
With unremitting pain—no moment free
In all thy day, thy night one prayer for rest?
Hope dead within, or languishing; and all
Thy future wrapped beneath a cold, grey pall?
Seems it as if all other lives were bright
With sunny beams that never shine on thee?
As if all other pathways led through light,
While only thine the vale of tears must be?
Though still in heart thou ownest him all just
The cry, "It is not fair," would mar thy trust?
For there are many blessings even for thee,
New every morning. Watch, and they will come
From Him Whose sweet compassion fails not. He
Will call thee when 'tis time to rest at Home.
Till then be thou content to do His will,
And thy poor heart shall grow resigned and still.

—*Prevost.*

**Reading.**

The position in which your Lord has placed you affords you precisely the means of grace and of succour adapted to the duties, troubles, and difficulties which meet you there. Give yourself up trustfully to God's Providence, which will require nothing of you for which He does not provide means. It is your part to ask and desire habitually that His holy will may be fulfilled in you, and through you, for those you love. However perplexing the way may seem now, rest satisfied that He will gradually unravel so much of it as you need from day to day, in order to follow His leading. You will never be really left without guidance. These trials are to you the royal road of the Cross; let each one be to you as a station at which you prostrate yourself in humble adoration of your Father's will. The last station will be heaven's very gate, and there you will be able to joy over your patient, faithful, quiet journey. If the path be sometimes dim, be like the little child, who, half-frightened in a twilight walk, clutches convulsively at its father's hand for protection. Consent freely that your wishes, thoughts, intentions, affections, all that is in you and of you, be reduced to the proportions of the cross chosen for you by God; for it is thus only that you can be made one with Christ, and in such oneness with Christ crucified you will find strength, light, comfort, and cure for all your doubts and difficulties; your heart's sore places will be soothed and healed. Fix your eyes on Him hidden beneath the Sacramental veil, and be strong in the assurance that these troubles, bravely borne now, will be your great joy hereafter. He is looking lovingly upon you. Strive to keep yourself in an attitude of entire resignation, and for that end do not scruple to pour out to Him all the heaviness, and even bitterness, which in spite of yourself floods your heart, and He will give you a strength you never dreamt of.—*From "The Light of the Conscience."*

Art thou patiently toiling, waiting the Master's will,

For a rest that never seems nearer, a hush that is far off still?

Does it seem that the noisy city never will let thee hear

The sound of His gentle footsteps, drawing, it may be, near?

Does it seem that the blinding dazzle of noonday glare and heat

Is a fiery veil between thy heart and visions high and sweet?

What though a lull in life may never be made for thee?

Soon shall a better thing be thine—the Lull of Eternity!

—*F. R. Havergal.*

### Notes and Queries.

**The Editor is always willing to answer in this column, without any fee, all reasonable questions, as soon as possible.**

**But the following rules must be carefully observed:—**

**1. Every communication must be accompanied by the name and address of the writer.**

**2. The question must always bear upon nursing, directly or indirectly.**

**If an answer is required by letter a fee of half-a-crown must be enclosed with the note containing the inquiry.**

*Probationer.*

(131) "S. P." would be glad to know where, at the age of 21, she may get a few months' training in a children's hospital.

The only way is to apply to the matrons of institutions which accept probationers of this age, stating height, health, &c. For lists see the "Nursing Profession: How and Where to Train."

*School.*

(132) Will you kindly inform me of a home or school for boy of ten years? Free, or moderate payment could be made. Mother a nurse, father incurable.—*Nurse U.*

Could you see a copy of Burdett's "Hospitals and Charities"? There are a large number of educational charities, and you would be able to select from the list given there those most suitable for your purpose.

*Eczema.*

(133) Will you kindly tell me the best thing to use for eczema? I am nursing an elderly gentleman who suffers very much, but nothing seems to ease him. Is there any cure for eczema? I should be glad to know.—*Nurse L.*

Eczema is very difficult to deal with, and must be treated by a medical man.

*India.*

(134) Will you kindly tell me how I can obtain a post in India as a midwife and dispenser. I am a qualified nurse as well.—*Dispenser.*

The Up-Country Nursing Association, The Cedars, Ealing, or the Zenana Medical and Bible Mission, 2, Adelphi Terrace, W.C., might possibly be glad to avail themselves of your services.

*South of France.*

(135) Will you kindly tell me the addresses of some of the institutions for private nursing in the South of France, especially those that need nurses for the winter months.—*C. W. M.*

The Hollond Institute, 1, Tavistock Chambers, Bloomsbury, W.C.; applications to be made in October only, when an advertisement appears in "Mirror." The English Nurses' Home, Châlet Marcelle, Biarritz; applications in July and August.

*Holidays.*

(136) I have been here twelve months on July 1st, and most likely will be leaving here about October next, though not for certain. Do you think it right of me when offered my holiday to take it?—*Fair Play.*

Under the circumstances you would be quite justified in taking your holiday when due.

*Egypt.*

(137) I am exceedingly anxious to go to some place in the north of Africa, Egypt by preference. I hold a three year certificate from the Glasgow Royal Infirmary, and have a knowledge of French, German, and Spanish. I should prefer private work.—*Buscando.*

Private nursing is difficult in Egypt because of the expenses of living. You might apply to Miss Bruce, matron of the Kasr-el-Aini Hospital, Cairo, as she has some English nurses on the staff, and to Miss Cutler, Victoria Nursing Home, Cairo. Miss James has also a nursing home in Cairo, and there is a nurses' home at Alexandria. There are numerous private homes in other places, and you might learn particulars by writing to the English consuls or chaplains.

*Deafness.*

(138) I have been asked by a lady what will cure her of slight deafness and loss of taste, the result of having had influenza. Can you tell me?—*Cordellia.*

We cannot prescribe.

*Maternity.*

(139) Will you kindly tell me if the training at the Edinburgh Royal Maternity Hospital ranks with the training at the various London schools of midwifery?—*Nora.*

Yes. The course of training is for three months, the number of probationers is limited, and the experience offered ample.

*Addresses.*

(140) 1. Will you kindly tell me the name and address of the secretary of the Up Country Nursing Association for India? 2. Will you also kindly tell me the name and address of the lady in Hertfordshire (mentioned in The Hospital recently) who makes marmalade suitable for invalids?—*Devonshire.*

1. The Cedars, Ealing, W. 2. The lady who makes "Parochial" mamalade lives at King's Langley.

**Standard Books of Reference.**

**"The Nursing Profession: How and Where to Train." 2s. net.**
**"The Nurses' Dictionary of Medical Terms." 2s.**
**"Burdett's Series of Nursing Text-Books." 1s. each.**
**"A Handbook for Nurses." (Illustrated.) 5s.**
**"Nursing: Its Theory and Practice." New Edition. 3s. 6d.**
**"Helps in Sickness and to Health." Fifteenth Thousand. 5s.**
**All these are published by The Scientific Press, Ltd., and may be obtained through any bookseller or direct from the publishers, 28 & 29, Southampton Street, London, W.C.**

### Travel Notes.

### LII.—THE ISLE OF MAN.

I PROPOSE this week to take into consideration a place suitable as a holiday resort for those whose home is in the North of England and who do not wish to go abroad. Naturally, the expenses of living in the Isle of Man will be considerably higher than in many parts of France, Switzerland, or Belgium. Alas! it is so, and hotel keepers have not yet awakened to the fact that their high charges are sending more and more of their clients away from them, to the lands where a holiday does not swallow up the savings of a whole year. However, I must in fairness say that providers of accommodation in the Isle of Man are by no means so rapacious as many in our own island.

### The Journey and its Expenses.

You can reach Douglas, the capital of the Isle of Man, from several different spots. Liverpool, Fleetwood, and Barrow all send steamers across, and from Ireland one can go over viâ Dublin and Belfast, whilst Scotch folk can cross from Glasgow, Ardrossan, or Sillioth. In the tourist season very cheap trips are arranged from London, beginning with one at 22s. for three days. The ordinary return ticket from London is 68s. 6d. first-class, and 54s. second, whilst a third only costs 38s. 6d.

### An Excellent Country for Cyclists.

The island being very small, a cyclist, if he establishes himself somewhere about the middle, may see the whole country with ease. If time and means admit of it, the best plan would be to spend a few days at Douglas, cycling up and down between Castletown in the south, and Ramsey in the north, taking in all the lovely country round Laxey; then removing to Ramsey and scouring the country all round the north, and as far south-west as Peel; finally taking up your quarters at Peel itself, and thoroughly doing the rest of the island south round by Port Erin, Port St. Mary, and Castletown. There is a very good cyclists' map of the country published by Brown and Sons, Athol Street, Douglas, by which you will see how easy it would be to see the whole island in ten days on your bicycle, though it would be far pleasanter not to be so driven, and to feel that you had a month at your disposal.

### Expenses of Living.

The hotels are reasonable, averaging 7s. 6d. per day, unless you want the best rooms. Apartments also are not dear (for the British Isles), and in comparatively quiet places like Port St. Mary lodgings may be had at 2s. per day for one room with attendance.

### Douglas.

This is naturally the gayest part in which to stay, and has the advantage of being a splendid centre, though personally I prefer Laxey, a little to the north, of which I will speak presently. When one has anything to sell there is nothing like vaunting one's goods with a loud trumpet; modesty is quite out of place. This opinion is shared by an inhabitant and citizen of Douglas. Hear what he says: "To attempt to describe the attractions of Douglas is like attempting to gild refined gold. There is not a seaside resort in the kingdom which can show anything like the advantages and the attractions which Douglas can show." I always respect one whose trumpet gives forth no uncertain sound. Indeed, it is true that distractions abound in the bright little town. Bowling, golf, and cricket for the active-minded, good bands playing all through the season for the pleasure of the leisurely, a very good theatre, and a succession of dances given at the Palace and the Derby Castle for the gaily-disposed. Boating and bathing are both excellent, and the excursions to be made in the neighbourhood are endless. The electric tramway and the railway companies have so arranged matters that the whole island is thrown open to the adventurous traveller.

### Laxey.

About five miles north is Laxey, one of the most beautiful spots in the island, in the midst of lovely wooded scenery, picturesque waterfalls, and sylvan delights of all sorts. Snaefell is distant about five miles from Laxey, perhaps hardly so much, and is reached by the electric tramway. The transit is ideal; one gradually ascends over 2,000 feet, and every inch is beautiful. A very fine spot on the road between Laxey and Ramsey is The Dhoon, where there is a really good waterfall.

### Peel.

On the western side of the island lies Peel. If you do not stay there it is easily visited from Douglas. The old town and castle, of which only the ruins now remain, are full of interest. Devout readers of Sir Walter Scott will remember the incidents in "Peveril of the Peak" which took place at the Sodor or Holm-Peel, as the castle used to be called. Its appearance in the time of Charles II. must have been most imposing and its position almost impregnable. Immediately under its walls is the old church of St. Germain, and according to Sir Walter there was another dedicated to St. Patrick still standing in the seventeenth century. The town of Peel looks charmingly picturesque from the harbour, and makes a delightful subject for an artist.

### Castletown, Port Erin, and Port St. Mary.

Castletown does not take my fancy so much, but Port Erin and Port St. Mary are both fascinating. Port Erin is quite a gay little place in a modest way. Boating, bathing, and fishing go on merrily, and excursions near and far are plentiful. It is a mild place, and more relaxing than Ramsey and Douglas. Port St. Mary was until lately a modest little fishing village, and still retains much of its old character round the harbour. I prefer it myself to Port Erin; the Mull hills afford admirable walks, and the wild coast scenery round the "Calf" is full of interest to a good pedestrian; indeed, your achievements in that line need not be great, for I think a walk of five miles would carry you all round the "Calf." I have left myself no room to speak of Ramsey. I do not think it such a good centre as Peel, because it is so much further north, but it has its own attractions, and had I more space I should like to have told you more about it.

---

### TRAVEL NOTES AND QUERIES.

Rules in Regard to Correspondence for this Section.—All questioners must use a pseudonym for publication, but the communication must also bear the writer's own name and address as well, which will be regarded as confidential. All such communications to be addressed "Travel Editor, 'Nursing Mirror,' 28, Southampton Street, Strand." No charge will be made for inserting and answering questions in the inquiry column, and all will be answered in rotation as space permits. If an answer by letter is required, a stamped and addressed envelope must be enclosed, together with 2s. 6d., which fee will be devoted to the objects of the "Hospital Convalescent Fund." Any inquiries reaching the office after Monday cannot be answered in "The Mirror" of the current week.

By Sea or Land to St. Andrews (M.P.)—The ordinary third-class ticket to St. Andrews is 36s. 4½d. Return for two months, 69s. 5d., but there are cheaper tourist tickets at that season. The nearest port to disembark is certainly Dundee; Glasgow is so very far away. As to expense, I think the sea passage would be the dearest, as it takes a long time. I have not my shipping lists with me, being on the continent, so cannot speak with certainty. Send a stamped and addressed envelope to Messrs. Gaze and Sons, Tourist Agents, 142, Strand, and they will give you all information. You can use my name if you like.

Orkney and Shetland (Matron).—It is not in our line to keep lists of lodgings in different places. I should recommend you to go to an hotel for the first day, and look about you quietly. I fear lodgings do not abound, though there are certainly some. With regard to hay fever take a doctor's advice. It would appear to be a favourable place, being surrounded by sea so much, but there are other considerations. Some cases find themselves distinctly worse in the vicinity of the sea.

